# Implementing tokenization in clinical research to expand real-world insights

**DOI:** 10.3389/fdsfr.2025.1519307

**Published:** 2025-02-13

**Authors:** Chelsea Walters, Crystal S. Langlais, Eva E. Oakkar, Wilhelmina E. Hoogendoorn, James B. Coutcher, Mui Van Zandt

**Affiliations:** ^1^ Real World Solutions, IQVIA, Durham, NC, United States; ^2^ Department of Epidemiology and Biostatistics, University of California, San Francisco, San Francisco, CA, United States

**Keywords:** tokenization, de-identification, data linkage, data privacy, real-world data, real-world evidence

## Abstract

Interest in leveraging real-world data (RWD) to support clinical research is increasing, including studies to further characterize safety and effectiveness of new treatments. Such studies often require a combination of primary, study-specific data, with secondary, existing RWD. So-called enriched studies are becoming more common but require tailored methodologies that facilitate linkage across data sources. Tokenization has emerged as a key tool in the United States (US) to enable the linkage of secondary data with primary data, although key considerations to operationalize tokenization are often overlooked during study set-up. This article aims to explore key aspects for implementing tokenization in the US and to define relevant terminology. Appropriate study designs and RWD sources to leverage this tool are also discussed and advantages and considerations for study stakeholders to enhance possibilities to generate real-world evidence are highlighted. The article concludes with a description of case studies where tokenization is a suitable fit to fulfill study goals.

## Introduction

Regulatory agencies are encouraging the broader use of real-world data (RWD) to draw further insights on safety, effectiveness, and use of drugs and other pharmaceutical products and medical devices (US Food and Drug Administration. Guidance for Industry, 2024; US Food and Drug Administration. Draft Guidance for Industry and FDA Staff, 2023; Klonoff, 2019). This interest has triggered an increasing need to combine data collected for study purposes (primary data) with existing (secondary) RWD, yielding an enriched dataset, while maintaining data privacy. This approach capitalizes on the advantages of deriving insights from both primary and secondary data. Specifically, narrowing primary collection to data that are challenging to capture from alternative sources can ensure high data quality while minimizing participant and research site burden. Leveraging secondary data can enable many cost-effective benefits, such as longer follow-up, contextualization of clinical trials data (e.g., external comparator designs), and providing a more holistic view of the participant journey and outcomes. Numerous secondary data sources comprised of real-world, participant-level data are available and may be considered fit-for-purpose for a given study. These include various US registries (e.g., state cancer registries; Surveillance, Epidemiology, and End Results [SEER]; National Cancer Registry; National Death Index), site- or systems-based electronic medical records (EMRs), claims data, and pharmacy prescription data. There have also been use cases linking non-medical secondary data (e.g., wearable devices). Using these existing data allows study sponsors to address more questions within a single study and such efficiencies help to accelerate the pathway to product approval and ultimately patient access. There are also sponsors who implement clinical trial tokenization without a definitive research objective. The rationale is to ensure that participant consent and personally identifying information (PII) have been collected to provide an option for future data linkage. Obtaining participant consent and/or PII collection after participant trial enrollment can introduce logistical and financial consequences.

Traditional approaches (i.e., those that leverage direct linkage of data sources) to linking participant-level data to address research objectives require sharing direct identifiers, such as personally identifying information such as PII, or availability of a common ID across data sources, which is often not feasible. Alternatively, tokenization of participant-level data allows for privacy preserving record linkage by converting PII into irreversible, hashed tokens based on multiple combinations of the input fields, ensuring the PII itself is not shared across institutions. Key to the success of any enriched study that utilizes tokenization will be identifying the right fit-for-purpose data, ensuring the appropriate tokenization engine is employed, proper planning to enhance privacy and minimize risk of re-identification, and ensuring proper consent from study participants is obtained. Published tokenization papers discuss broad uses, such as the effectiveness of using tokens in place of PII (Bernstam et al., 2022), or challenges and benefits specific to linking to trial data (Eckrote et al., 2024), but do not examine clinical operation considerations for study setup and execution. This article aims to inform the reader about tokenization by defining relevant tokenization terminology and providing key considerations intended to support successful implementation of a study that requires linking study data to RWD through tokenization. Case studies are used to aid discussion of study designs appropriate for tokenization, demonstrate nuanced considerations, and highlight advantages and considerations for study sponsors to enhance their research investment.

This article focuses on aspects of tokenization in the US. The maturity of tokenization usage in US RWD results from its unique fragmented healthcare system where numerous providers, payers and state governments are allowed to share or commercialize data in de-identified datasets. This creates a demand for solutions, including the linkage of such datasets through a privacy preserving methodology (i.e., tokenization). In the European Union (EU), the national healthcare systems that are prevalent generate a rich longitudinal data source, rendering commercially de-identified datasets less essential. In addition, the Internal Review Board (IRB) regulations regarding participant consent to data linkage (e.g., purpose of linkage, duration of PII retention) vary by country and these regulations fall under General Data Protection Regulation (GDPR). Interpretation and practice of the privacy considerations for EU tokenization use remain unclear.

## Tokenization process and key terminology

When planning tokenization for data linkage studies, there are key processes that need to be set up throughout the study lifecycle. In the tokenization process, PII is captured and converted to track the same participant across data sources. PII is “any representation of information that permits the identity of an individual to whom the information applies to be reasonably inferred by either direct or indirect means” (e.g., PII includes first name, last name, date of birth, address, etc.) (Ferraiolo et al., 2024). Of note, personal health information (PHI) encompasses all medical and demographic records of a participant in the healthcare setting or a participant in a study.

The tokenization process facilitates linkage of multiple datasets by converting PII into a secure, irreversible string of characters known as tokens. Using different combinations of PII inputs, multiple tokens can be assigned to the same participant to increase statistical accuracy of matching to the correct participant across data sources. For example, one token could be a combination of first name, last name, and street address, and another token could be a combination of first name, last name, gender, and zip code (Bernstam et al., 2022). These tokens allow for de-identified linkage of the various datasets to generate longitudinal participant data.

De-identification is a general term for any technique that reduces the identifiability of the participant represented in a dataset. The term stems from the US Health Insurance Portability and Accountability Act (HIPAA) of 1996, which notes that de-identification yields data that “neither identifies nor provides a reasonable basis to identify an individual” (US Department of Health and Human Services, 2022).

Anonymization and pseudonymization are also often discussed during the tokenization process to ensure participant data privacy. Anonymization is a sub-category of de-identification where the applied technique renders the resulting datasets irreversibly non-identifiable. Traditional study data are commonly pseudonymized via a participant study ID, wherein an individual cannot be identified from the study database, but a standalone linkage code (stored in a secure location and separate from the study database) would allow personnel with access to the linkage code to determine the individual’s identity.

When linking clinical study data with RWD via tokens generated from PII, it is important to note that while the token is de-identified, the dataset that results from clinical study data and RWD linkage must be de-identified as well. This is accomplished with a re-identification risk determination (RRD) analysis which is further described in the below section: “Re-identification Risk Determination and Transformation of Data Fields.”

## Considerations during study start up

### Consent capture and withdrawal of consent

Participant consent is typically required for PII collection, tokenization, and data linkage. Appropriate language should be included in the informed consent form (ICF) at study start-up, including the known and intended purposes for tokenization. As a note, in some instances a study sponsor can pursue a waiver of consent from the IRB approving the study, if the relevant criteria are fulfilled (US Department of Health and Human Services, 2021), while participants in interventional trials typically need to be allowed the option to consent to the research study while opting out of having their data tokenized. Additionally, processes and procedures need to be established to ensure withdrawal of consent is handled properly.

After obtaining consent, the PII of study participants is collected into a secure portal (separate from other study data) by enrolling sites or by the participants directly. If the study sponsor is looking to tokenize and link the data in the future, PII can be stored until the linkage requirements are defined. In addition, data use agreements should be signed between the sponsor and data source holders to be able to link to those data sources in the future and understand any restrictions (such as de-identification). However, a limitation of this so-called “future proofing” using tokenization is that there is a certain level of expiration to tokens, including the case where PII changes over time (name changes, address changes, etc.).

Once the data linkage is defined, the PII is sent to a secure repository where the tokenization engine is housed. The tokenization engine uses the collected PII from study participants to generate the token of choice. Typically, the sponsor is sent generated tokens with the corresponding study ID of the participant so the token can be attached to the study (primary) data.

### Fit-for-purpose data and need for a feasibility study

A key tenet of the US Food and Drug Administration (FDA) Real-World Evidence (RWE) framework is identifying fit-for-purpose data (Gatto et al., 2022). Fit-for-purpose data means the data sources are relevant and reliable and are suitable to address the objectives of the study. The FDA’s draft guidance on the *Use of RWE to Support Regulatory Decision-Making for Medical Devices* provides insights as to the types of assessments that may be warranted to determine a fit-for-purpose data source (Gatto et al., 2022; US Food and Drug Administration. Draft Guidance for Industry and FDA Staff, 2023). Other authors have provided more detailed guidance on performing fit-for-purpose assessments (Gatto et al., 2022; Duke Margolis Institute for Health Policy, 2019). Briefly, assessing the *reliability* of data requires evaluating data quality, accuracy, integrity, and completeness, as well as whether the data adequately capture the underlying concepts relevant to the study. Assessing the *relevancy* of data requires evaluating whether the given data can address the research objective. Further assessments are also warranted to evaluate data access, including timeliness, permissions, and linkage probabilities.

Assessing whether data are fit-for-purpose is an important step before choosing the RWD sources to be utilized; findings from these assessments will inform the feasibility of a given study. The importance of this assessment should not be ignored. Especially when data will be submitted for regulatory purposes, sponsors should consider a feasibility study as a first step. In addition, data linkage through tokenization should only be applied after it is determined that the RWD sources are indeed fit-for-purpose and the linkage rate is adequate. When assessing fit-for-purpose, the sponsor should consider how much of the data can be preserved after linkage (e.g., the RRD might recommend some data being nulled [i.e., suppressed/deleted], etc.) and if the data are still valuable to evaluate study outcomes after data transformations based on an RRD are applied.

## Implementation considerations

### Tokenization versus direct linkage

An advantage of tokenizing study datasets is that study sponsors do not need to link to other datasets immediately. By capturing consent and the capability to potentially link to other data, use cases can remain undefined until the need arises while avoiding the challenges of obtaining consent for tokenization retroactively. Consent for tokenization and data linkage can be included in the main study consent or managed as a separate consent, if data linkage is not crucial to participant enrollment and study conduct. Although “future proofing” is possible through capturing participants’ consent and PII, knowing the evidence needs when planning a real-world study or clinical trial is preferable as it allows for other data linkage options beyond tokenization, such as direct linkage, which may better address the research objectives. Direct linkage of datasets does not use tokens, but instead employs other identifiers (e.g., participant study ID) to link different data sources, such as study electronic data capture (EDC) data to a participant’s EMR (if study ID is being tracked in the research sites’ EMR systems). An example of direct linkage is explained more in detail in the “Best Fit User Cases for Tokenization” section below.

Tokenization is often the linkage method of choice when the secondary datasets of interest are unknown at the time of conducting the study (not allowing for real-time direct linkage) or when the relevant data sources are de-identified. A common use case is healthcare resource utilization (HCRU) analyses since most claims data sources are de-identified and difficult to link by other methodologies. In such cases, direct linkage would not be possible since it uses participant identifiers, such as a participant study ID, to link to the selected data source and, thus, could lead to potentially re-identifying participants in de-identified data sources.

### Choosing a tokenization engine

Multiple tokenization engines exist which enable linking to certain datasets. Token vendors have established an ecosystem of data sources with their tokens. The tokenization engine that best fits a study’s needs is determined by the tokens used in the existing, secondary data sources of interest. Once participants’ consent and PII are collected in a study, tokens can be created with any one or multiple tokenization engines. If needed, crosswalk tables are available which connect a participant’s tokens from one token vendor to the same participant’s tokens from another token vendor. It is important to note that using a token from a crosswalk table is often less statistically accurate than generating that same token directly from source PII data.

## Considerations during study conduct

### Re-identification risk determination and transformation of data fields

Participant privacy is of high importance in any study. When two or more datasets are combined, the combination of the data in the resulting dataset may allow for indirect identification of individuals. Thus, managing participant data privacy throughout the tokenization lifecycle requires special attention, especially when at least one of the datasets has a requirement to remain de-identified. Study (primary) data are typically identifiable and need to undergo a de-identification process before combining the datasets to ensure the resulting combined dataset remains de-identified. An RRD is an important tool in such use cases. RRD is an analysis that is completed before linkage occurs to determine if the linked dataset will remain de-identified in accordance with the HIPAA Privacy Rule’s Expert Determination standard. In other words, RRD is an assessment to predict the effect that linking the dataset will have on the risk of re-identification. RRDs are performed using generally accepted statistical methods in accordance with applicable data protection laws. If the analysis concludes that the linked dataset would not be de-identified, then risk mitigation strategies need to be implemented. Examples of mitigation strategies include masking data fields (e.g., replace raw age with age categories), shifting date fields using a random offset, and nulling data fields. These modifications act to preserve participant privacy. It should be noted that as more datasets are linked together, the RRD analysis becomes increasingly restrictive to preserve participant privacy and thus, more transformations might need to be applied to the linked dataset. Additionally, it may be necessary to use separation of environments to ensure privacy-preserving linkage.

## Best fit use cases for tokenization

Many use cases are available that have demonstrated the utility of tokenization. Two case studies are highlighted here as illustrative examples.

### Known purpose for tokenization through linking de-identified datasets

This case study required linkage of multiple data sources to evaluate the use and adherence of the drug in the study arm versus an external control cohort. Although the study arm utilized primary data sources (e.g., participant-reported outcomes [PROs]), all endpoint data for both groups came from secondary data sources, which included claims and EMR from integrated delivery networks (IDNs). These secondary data sources were linked with multiple primary data sources (participant-reported outcomes, safety data, study drug distribution and discontinuation data).

As a first step, a feasibility study was performed to determine which RWD were fit-for-purpose. As part of this feasibility, the linkage rate, data completeness, and availability of critical data were assessed. Findings from this feasibility study yielded a decision to proceed and informed the full study design, including identification of the most appropriate data sources to use.

The consent form reflected the intention to collect, tokenize, and link data. Participants identified in the external control cohort were able to be included under a waiver of consent since no primary data was being collected. A secure portal (separate from all study data) was set up to house the PII, and these data were eventually sent to a secure repository where the tokenization engine was housed.

The tokenization algorithm required first name, last name, date of birth, gender, street address, and zip code. The first four of these fields have been shown to have the highest precision for data matching (Bernstam et al., 2022). Resulting tokens were matched to existing secondary data sources using a proprietary matching software.

To protect participant privacy prior to linking multiple datasets, an RRD analysis was completed. This analysis identified the need for different data transformations, including masking untransformed direct identifiers from all data sources (e.g., study ID), nulling some diagnoses codes, and generalizing age for those greater than 85 years. A more complicated RRD transformation that was recommended was date shifting certain date fields (e.g., hospital admission and discharge dates) across data sources. To avoid errors in the analysis, coordination across datasets was needed to establish a consistent date shift of all date fields on a given participant.

Following the finalization of the RRD analysis, a trusted third party (TTP) received and handled multiple identifiable datasets and PII and used the RRD analysis to de-identify these datasets. In addition, the TTP created a series of irreversible, hashed tokens. These tokens were sent back to the study sponsor, along with the de-identified datasets, to allow for data linkage and endpoint analysis.

### Use of tokenization to validate past study data

In this use case, a study sponsor was interested in evaluating participant diversity after enrollment had concluded. PII and consent had been collected as part of clinical study operations. Tokens were created with an algorithm requiring first name, last name, date of birth, gender, street address, and zip code and then matched to existing secondary data sources using a proprietary matching software.

Racial and ethnic data from a consumer credit reporting company database were linked to study data using tokenization. Through this approach the study sponsor was able to demonstrate that study participants met the diversity goals. The sponsor elected to limit the consumer data linked only to those fields to address diversity evidence needs. This allowed for better retention of clinical data following the RRD. Without the ability to link through tokenization, the primary study may have had to be repeated, resulting in additional time and cost. This case study is one example of how tokenization can be utilized to fulfill a data need that was undefined at the study start.

### Future potential

Tokenization may potentiate further creative solutions yet to be realized. Leveraging RWD using tokenization offers further promise for additional insights into long term follow-up of study participants and enhanced capture of participant status in lost to follow-up.

## Discussion

Tokenization allows for clinical research (primary) data to be linked to RWD, streamlining insight generation. There are key considerations to both setting up and implementing tokenization, such as including the appropriate tokenization language in the ICF, managing withdrawal of consent, identifying the appropriate tokenization engine(s), and assessing linkage rates. When using tokens to link multiple datasets, an RRD analysis is recommended to preserve participant privacy. Tokenization for both clinical trials and real-world studies offers promise, but the appropriate fit-for-purpose linkage method must be determined on a case-by-case basis. When applied to appropriate use cases, tokenization can be utilized to facilitate opportunities for evidence generation across the pharmaceutical lifecycle.

## Data Availability

The original contributions presented in the study are included in the article, further inquiries can be directed to the corresponding author.
